# Supplementary material for: Role of Metformin in Morbidity and Mortality Associated with Urinary Tract Infections in Patients with Type 2 Diabetes
Source: J Pers Med. 2022 Apr 28;12(5):702. doi: 10.3390/jpm12050702 (PMC9144588; doi:10.3390/jpm12050702)
Supplement: Supplementary file 1 [file jpm-12-00702-s001.zip › jpm-1679349-supplementary.pdf]

Supplementary Table S1. Diseases and related ICD-9-CM, ICD-10-CM codes.

Disease	ICD-9-CM Codes	ICD-10-CM Codes
Type 2 diabetes mellitus	250.xx, except 250.1x	E11
Type 1 diabetes mellitus	250.1x	E10
Hepatic failure	570, 572.2, 572.4, 572.8	K72.00, K72.01, K72.10, K72.11, K72.90, K76.2, K72.90, K72.91, K76.7, K76.81
Surgery involving the urinary system	55-59	0T
Malignant neoplasm of the urinary tract	179-189	C64-C68
Malignant neoplasm of lymphatic and hematopoietic tissue	200-208	C81-C96
Dialysis	V56.0, V56.8, V45.1	Z49.31, Z49.32, Z99.2
Overweight	278.02, 783.1, V85.2	R63.5
Obesity	278.00, 649.1, V77.8, V85.3	E66.09, E66.1, E66.8, E66.9, Z13.89
Severe obesity	278.01, 649.2, V45.86, V85.4	E66.01, E66.2
Smoking status	305.1, 649.0, V15.82	F17.200, F17.201, F17.210, F17.220, F17.221, F17.290, F17.291, Z87.891
Alcohol disorders	291, 303, 305.0, 571.0-571.3, V11.3, V79.1	F10, K70.40, K70.41, K70.9
Hypertension	401–405, A26	I10, I11, I12, I13, I15, N26
Dyslipidemia	272	E71.30, E71.31, E71.32, E71.39, E75.21, E75.22, E75.23, E75.24, E75.25, E75.29, E75.3, E75.4, E75.5, E75.6, E77, E78.0, E78.1, E78.2, E78.3, E78.4, E78.5, E78.6, E78.70, E78.71, E78.72, E78.79, E78.8, E78.9
Coronary artery disease	410-414	I20, I21, I22, I24, I25.1, I25.2, I25.3, I25.4, I25.5, I25.6, I25.7, I25.81, I25.82, I25.83, I25.84, I25.89, I25.9
Chronic kidney disease	250.4x, 403.xx, 404.xx, 585.xx, 586.xx, 581.8x, 791.0x, 593.9x, V42.0x, V45.1x, V56.0x, V56.8x, 39.27, 39.42, 39.43, 39.49, 39.50, 39.53, 39.93, 39.94, or 39.95	E10.2, E10.65, E11.2, E11.65, E13.2, I12, I13, N03, N08, E10.21, E11.21, N05, N06, N07, N14, N15.0, N15.8, N15.9, N16, N17.1, N17.2, N18, N19, Z94.0, Z49.31, Z49.32, Z99.2, Z94.0
Stroke	430-438	G45.0, G45.1, G45.2, G45.3, G45.4, G45.8, G45.9, G46, I60, I61, I62, I63, I65, I66, I67.0, I67.1, I67.2, I67.3, I67.4, I67.5, I67.6, I67.7, I67.8, I67.9, I68, I69
Atrial fibrillation	427	I45.0, I45.1, I45.2, I45.3, I45.4, I45.5, I45.6

Heart failure	398.91, 402.01, 402.11, 402.91, 404.01, 404.03, 404.11, 404.13, 404.91, 404.93, 428, 429.4	I09.81, I11.0, I13.0, I13.2, I50, I97.0, I97.110, I97.111, I97.120, I97.121, I97.130, I97.131, I97.190, I97.191
Peripheral arterial occlusion disease	440.0, 440.20, 440.21, 440.22, 440.23, 440.24, 440.3, 440.4, 443.9, 443.81, 443.89	I70.2, I70.92, I75.0, I73.9
Retinopathy	362.02, 362.07, 362.0	H35.0, E08.311-E08.359, E09.311-E09.359, E11.311-E11.359, E13.311-E13.359
Chronic obstructive pulmonary disease	491, 492, or 496	J41, J42, J44, J43, or J44.9
Rheumatoid arthritis	714.0	M06.9
Systemic lupus erythematosus	710.0	M32.10
Hepatitis B and C infection	277.4, 570, 572.8, 573.3, 573.8, 576.8, 782.4	B15.0-B19.9
Liver cirrhosis	571.5, 571.2, 571.6	K70.2, K70.30, K70.31, K74.0, K74.1, K74.2, K74.60, K74.69, K74.3, K74.4, K74.5
Urolithiasis	592, 594	N20-N23
Cancers	140-178, 190-199, 209	C00-C63, C69-C80, C7A-C7B
Psychosis	290–299, except 290,290.4, 291.2, 292.82	F20-29
Depression	311	F32, F33
Dementia	290,290.4, 291.2, 292.82 and 331	F03.90, F05, F02.80, F02.81, F01.50, F01.51, G30
Urethritis	597	N34
Cystitis	595	N30
Acute pyelonephritis	590	A10
Sepsis	790.7, 038, 041, 995.91, 995.92	R78.81, A41, R65.20, R65.21
